# Attenuation of ligand-induced activation of angiotensin II type 1 receptor signaling by the type 2 receptor via protein kinase C

**DOI:** 10.1038/srep21613

**Published:** 2016-02-09

**Authors:** Takayuki Inuzuka, Yoichiro Fujioka, Masumi Tsuda, Mari Fujioka, Aya O. Satoh, Kosui Horiuchi, Shinya Nishide, Asuka Nanbo, Shinya Tanaka, Yusuke Ohba

**Affiliations:** 1 Department of Cell Physiology, Hokkaido University Graduate School of Medicine, Sapporo 060-8638, Japan; 2 Department of Cancer Pathology, Hokkaido University Graduate School of Medicine, Sapporo 060-8638, Japan

## Abstract

Angiotensin II (AII) type 2 receptor (AT2R) negatively regulates type 1 receptor (AT1R) signaling. However, the precise molecular mechanism of AT2R-mediated AT1R inhibition remains poorly understood. Here, we characterized the local and functional interaction of AT2R with AT1R. AT2R colocalized and formed a complex with AT1R at the plasma membrane, even in the absence of AII. Upon AII stimulation, the spatial arrangement of the complex was modulated, as confirmed by Förster resonance energy transfer (FRET) analysis, followed by AT2R internalization along with AT1R. AT2R internalization was specifically observed only in the presence of AT1R; AT2R alone could not be internalized. The AT1R-specific inhibitor losartan completely inhibited both the conformational change and the internalization of AT2R with AT1R, whereas the AT2R-specific inhibitor PD123319 partially hindered these phenomena, demonstrating that the activation of both receptors was indispensable for these effects. In addition, treatment with the protein kinase C (PKC) inhibitors inhibited the ligand-dependent accumulation of AT2R but not that of AT1R in the endosomes. A mutation in the putative phosphorylation sites of AT2R also abrogated the co-internalization of ATR2 with AT1R and the inhibitory effect of ATR2 on AT1R. These data suggest that AT2R inhibits ligand-induced AT1R signaling through the PKC-dependent pathway.

Angiotensin II (AII) is a pleiotropic peptide hormone with crucial roles in the development of cardiovascular diseases, including hypertension, atherosclerosis, and heart failure^1^,^2^. To date, four distinct subtypes of AII receptors have been identified in humans. The type 1 receptor (AT1R) is the most studied receptor and is implicated in AII-induced vasoconstriction^1^,^2^. Extracellular signal-regulated kinase/mitogen-activated protein kinase (ERK/MAPK, hereafter referred to as ERK) is a key effector of the AT1R signaling cascade^3^. ERK is phosphorylated and activated by at least three distinct downstream AT1R signaling pathways, including the G-protein-coupled protein kinase C (PKC)-dependent pathway, the β-arrestin-dependent pathway, and the epidermal growth factor receptor (EGFR) transactivation pathway. Although the pathophysiological roles and signal transduction mechanisms of receptors other than AT1R are less clear, the AII type 2 receptor (AT2R) is reported to counteract AT1R-mediated actions^1^,^2^,^4^,^5^, in part through the inhibition of ERK activation.

AT2R expression in adults is restricted to a few tissues, including the brain and the uterus^6^. AT2R expression is increased in tissues undergoing remodeling resulting from hypertension, cardiac hypertrophy, and ischemic heart diseases^6^. Although the detailed molecular mechanism through which AT2R perturbs AT1R-induced ERK activation remains controversial, accumulating evidence suggests the participation of protein phosphatases, including mitogen-activated protein kinase phosphatase-1 (MKP-1), Src homology 2 domain-containing protein tyrosine phosphatase (SHP-1), and the serine-threonine phosphatase PP2A^7^,^8^. However, the direct binding of AT2R to AT1R has also been proposed as an alternative inhibitory mechanism of AT2R in AT1R signaling^9^. Such an interaction could be observed regardless of ligand binding. Because a chemical crosslinking method was used in that study, the precise regulatory mechanism of AT2R-mediated perturbation of AT1R under physiological conditions has not yet been determined.

Here, we utilized fluorescent protein-tagged AT1R and AT2R to identify a more physiologically relevant relationship between AT1R and AT2R and found that AT2R interacts with AT1R both *in vitro* and *in vivo*. The receptor complex was internalized by endocytosis in a manner dependent on the activities of AT1R, AT2R, and PKC. We also revealed that such internalization was associated with alterations in the relative orientations of AT1R and AT2R, in which their C termini were brought in close proximity to each other, by Förster resonance energy transfer (FRET) analysis. These findings shed light on the previously unknown molecular mechanisms of AT2R-mediated inhibition of ligand-induced AT1R activation.

## Results

### AT2R selectively inhibits AT1R-dependent ERK phosphorylation

**Figure 1:**
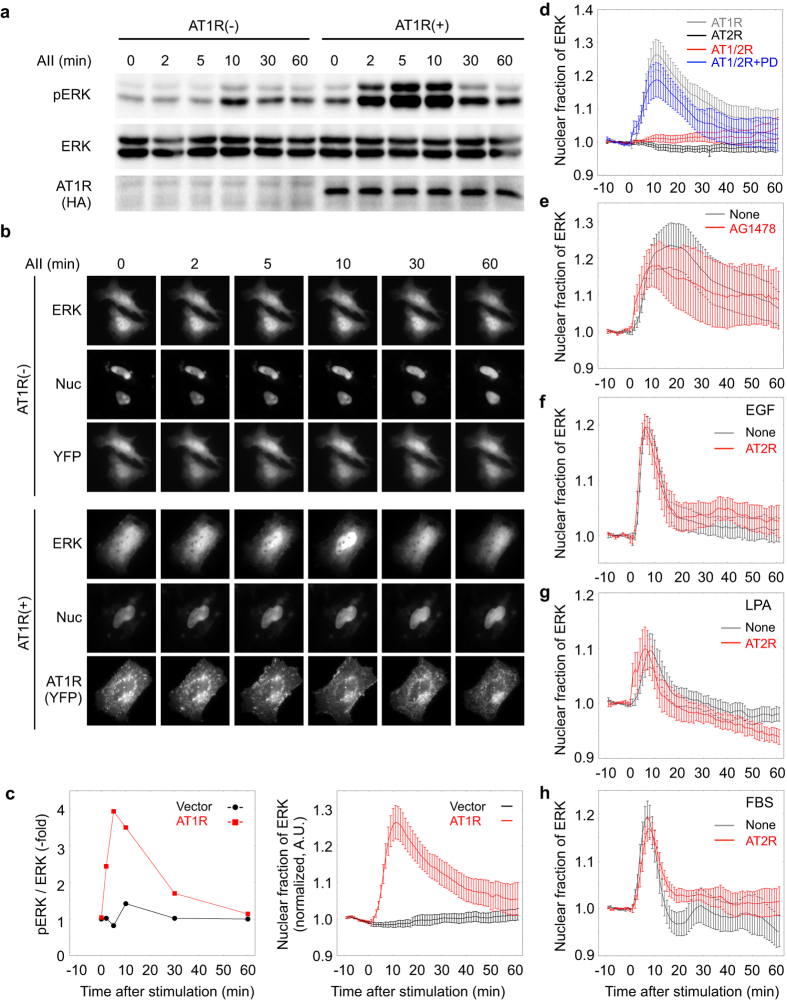
AT2R specifically disturbs AT1R signaling. (**a**) HeLa cells were transfected with an AT1R expression vector or a control vector. After 24 h, the cells were serum starved for 4 h and then stimulated by AII or left untreated. Cell lysates were collected at the indicated time points and then subjected to SDS-PAGE. The ERK phosphorylation status was determined by immunoblotting using a phospho-specific antibody against ERK. ERK and AT1R expression was also evaluated using antibodies against ERK and HA. (**b**) HeLa cells expressing eqFP650-ERK alone or together with AT1R were stained with Hoechst 33342 and monitored by epi-fluorescence time-lapse microscopy, with exposure to AII at time 0. (**c**) The intensity of the bands (in a; left panel) and the mean of the nuclear ERK/total ERK fluorescence intensities (in b; right panel) were quantitated and plotted. Error bars indicate the standard error of the mean (s.e.m.; n = 8 for time-lapse imaging). (**d**) HeLa cells expressing AT1R alone, AT2R alone, or both AT1R and AT2R were incubated in the absence or presence of the AT2R-specific inhibitor PD123319 for 4 h and were monitored by time-lapse fluorescence microscopy following exposure to AII at time 0. The mean nuclear ERK/total ERK ratio was plotted over time. Error bars indicate the s.e.m. (n = 5). (**e**) Cells expressing AT1R were incubated in the absence or presence of the EGFR inhibitor AG1478 for 4 h before stimulation with AII and were monitored by time-lapse microscopy. The mean nuclear ERK ratio was plotted over time. Error bars indicate the s.e.m. (n = 3). (**f–h**) HeLa cells transfected with the AT2R expression vector or the control vector were assessed by time-lapse microscopy in the presence of AII and were stimulated by EGF (**f**), LPA (**g**), and FBS (**h**) at time 0. Error bars indicate the s.e.m. [n = 6 (**f**), 5 (**g**), 5 (**h**)].

As AT2R signaling has been reported to counteract AT1R-dependent signaling^1^,^2^,^4^,^5^, we first examined the effect of AT2R on ERK phosphorylation induced by AT1R or other receptors. Whereas AII treatment activate ERK at negligible levels in naïve HeLa cells, it remarkably induce ERK phosphorylation in the presence of AT1R (Fig. 1a). Essentially similar results can be observed in human embryonic kidney 293T cells (Suppl. Fig. S1a). When we analyzed the localization of ERK2 tagged with the near-infrared fluorescent protein eqFP650 (ref. 10), we specifically observed AII-induced nuclear translocation of ERK in the presence of AT1R in HeLa cells (Fig. 1b). Because the time courses of ERK activation revealed by these methods were consistent with each other (Fig. 1c), we utilized eqFP650-ERK in the following experiments to monitor ERK activity in HeLa cells that expressed the intended proteins. HEK293T cells were also used to confirm the imaging data by biochemical analyses. Although AT2R expression failed to induce AII-dependent ERK phosphorylation, it attenuated AII-dependent, AT1R-mediated ERK activation (Fig. 1d; Suppl. Fig. S1a). This impairment of ERK phosphorylation was ascribed to AT2R signaling because treatment with PD123319, an AT2R inhibitor, restored AII-induced ERK activation (Fig. 1d).

We next investigated the selectivity of the AT2R-dependent attenuation of ERK activation. Treatment with the EGFR inhibitor AG1478 produced no inhibitory effects on AII-AT1R-dependent ERK activation (Fig. 1e; Suppl. Fig. S1b), showing that EGFR transactivation is dispensable for AT1R signaling in this cell line. Under this condition, we found that AT2R expression did not perturb ERK activation by factors such as EGF (Fig. 1f; Suppl. Fig. S1c), lysophosphatidic acid (LPA) (Fig. 1g; Suppl. Fig. S1d), and fetal bovine serum (FBS) (Fig. 1h; Suppl. Fig. S1e) that activate ERK via the ERK cascade [Raf/ MAPK-ERK kinase (MEK)/ERK]. Thus, these data indicate that AT2R selectively blocks AII-AT1R-dependent ERK activation and that the inhibition might occur upstream of the ERK cascade. In fact, the AII-AT1R-dependent membrane recruitment of c-Raf1, which is a hallmark of Raf activation, was inhibited by AT2R expression (Suppl. Fig. S2).

### AT2R interacts with AT1R

**Figure 2:**
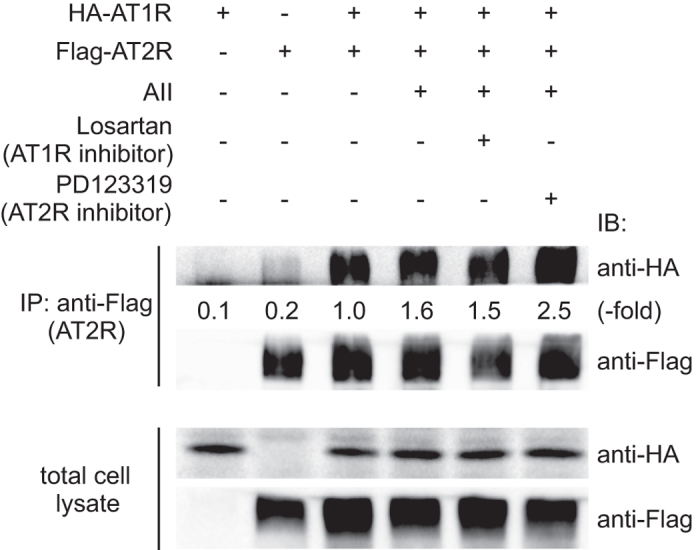
AT2R interacts with AT1R. HeLa cells transfected with the expression vectors indicated at the top were serum starved, pre-treated with the AT1R-specific inhibitor losartan or the AT2R-specific inhibitor PD123319, and stimulated by AII. The cells were lysed in lysis buffer and immunoprecipitated with an anti-FLAG antibody, followed by immunoblotting using an anti-HA or anti-FLAG antibody. An aliquot of total cell lysate was also analyzed by immunoblotting.

According to the results shown in Fig. 1, we hypothesized that AT2R attenuated AT1R signaling by forming complexes. In fact, the direct binding of AT2R to AT1R was previously demonstrated to occur by the immunoprecipitation method, and this interaction inhibited the G protein-coupled AT1R signaling pathway^9^,^11^. However, it remained unknown whether the interaction was enhanced by AII stimulation. Thus, we examined the direct interaction of AT2R with AT1R in our model cell line by using a co-immunoprecipitation assay. AT2R bound to AT1R, even in the absence of AII (Fig. 2, lane 3; Suppl. Fig. S3, lane 3), and AII treatment further enhanced the association between AT2R and AT1R by 1.6-fold in HeLa cells (Fig. 2, lane 4) and 2.4-fold in 293T cells (Suppl. Fig. S3, lane 4). However, treatment with losartan, an AT1R inhibitor, or PD123319 did not reverse this association enhancement. Therefore, the receptor interaction profile *per se* did not correlate with that of ERK activation shown in Fig. 1 and Suppl. Fig. S1. These results do not necessarily negate the possibility that AT2R perturbs AT1R signaling at the receptor level but rather suggest the requirement for approaches other than biochemical analyses to gain further insight into the signaling crosstalk mechanism.

### AII stimulation induces AT2R internalization in an AT1R-dependent manner

**Figure 3:**
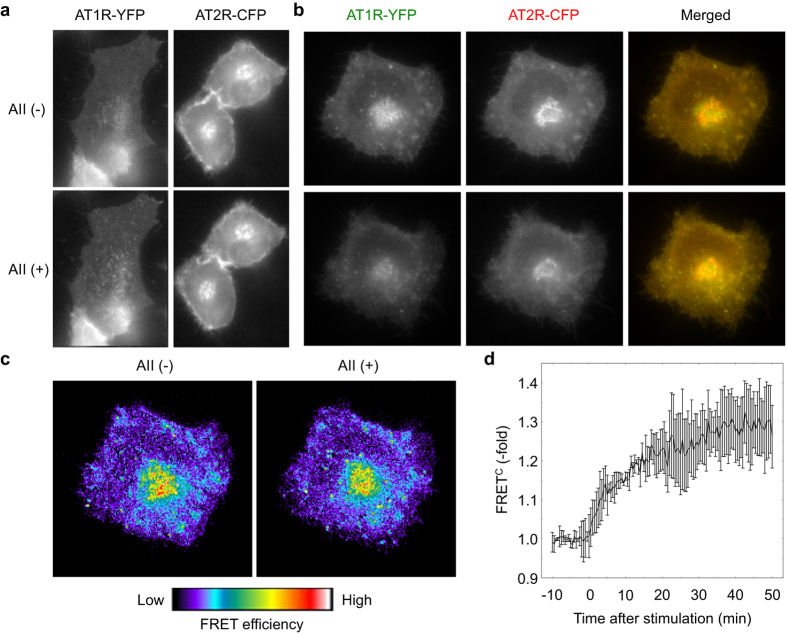
AT2R is internalized with AT1R. (**a**) HeLa cells transfected with expression vectors for AT1R-YFP or AT2R-CFP were observed with fluorescence microscopy. Images before and after AII stimulation (for 30 min) are shown. (**b**) HeLa cells expressing both AT1R-YFP and AT2R-CFP were subjected to multi-dimensional time-lapse fluorescence microscopy. After 10 min of recording, the cells were stimulated by AII. Merged images are shown at the bottom. (**c,d**) HeLa cells prepared as in (**b**) were imaged on a microscope, and the corrected FRET image (FRET^C^) was reconstructed and displayed in pseudocolor mode (**c**). The time course of mean FRET^C^ is also shown (**d**). Error bars indicate the s.e.m. (n = 3).

Because AT1R has been well documented to accumulate in the endosome upon AII stimulation^12^,^13^, we hypothesized that AT2R might participate in the regulation of AT1R signaling in a spatiotemporally distinct fashion. Therefore, to visualize the subcellular localization and trafficking of AT1R and AT2R, we prepared expression vectors for the receptors tagged with either cyan or yellow fluorescent proteins (CFP or YFP) and observed their localization. In the absence of AII, both AT1R and AT2R resided mainly at the plasma membrane (Fig. 3a). Upon AII stimulation, AT1R was immediately internalized, as described previously^12^,^13^, whereas AT2R was retained at the plasma membrane (Fig. 3a; Suppl. Mov. 1,2). We next examined the subcellular localization and changes in co-expressed AT1R and AT2R. Even in the absence of AII stimulation, the localization pattern of AT2R was comparable to that of AT1R (Fig. 3b), indicating that AT1R and AT2R colocalized; this finding was consistent with the co-immunoprecipitation assay results (see Fig. 2). However, upon AII stimulation, AT2R and AT1R were internalized (Fig. 3b; Suppl. Mov. 3–5), in contrast to what was observed in the cells expressing AT2R alone (Fig. 3a), and colocalized with the granular structures in which AT1R was localized.

FRET analysis is a powerful tool for studying molecular interactions in a living cell. Thus, we further analyzed FRET data for CFP-tagged AT2R and YFP-tagged AT1R. Whereas only a weak FRET signal was detected in quiescent cells (Fig. 3c), the signal was increased by AII stimulation (Fig. 3d), most apparently in the granular structures (Fig. 3c; Suppl. Mov. 6). The FRET signal remained high at least until 80 min after AII stimulation (Suppl. Fig. S4). These data collectively indicate that AT2R directly binds to AT1R, even in the absence of AII, but the molecular orientation does not bring CFP and YFP sufficiently close together for the FRET signal to be observed^14^. AII stimulation further enhanced the association between AT1R and AT2R and induced the conformational change that brought CFP and YFP close together, making the functional interaction between AT1R and AT2R possible.

### Ligand binding to both AT1R and AT2R is required to induce conformational changes in their complex

**Figure 4:**
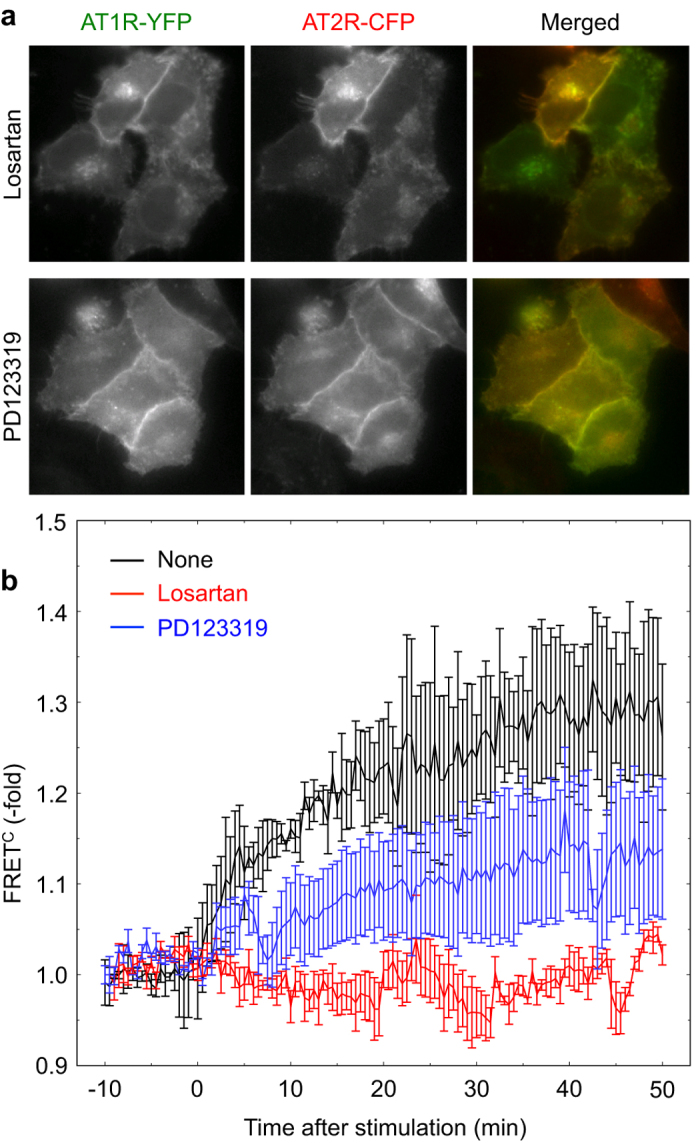
AII binding to the receptors is required for concomitant internalization. (**a**) HeLa cells prepared as described in Fig. 3c were pretreated with either losartan or PD123319. The cells were observed by fluorescence microscopy and then exposed to AII. (**b**) The cells prepared as in (**a**) were subjected to time-lapse microscopy. The time course of FRET^C^ in each sample was plotted. Error bars indicate the s.e.m. (n = 3).

To further clarify the relationship between AT1R and AT2R, we next examined the effects of receptor antagonists on the AII-induced association of the receptors. Treatment with losartan, an AT1R inhibitor, completely abolished the FRET signal between the two receptors as well as the internalization of both receptors (Fig. 4a,b), indicating that the AII-dependent internalization and conformational changes were entirely dependent on the AT1R signaling pathway. However, treatment with PD123319, an AT2R antagonist, did not hinder AT1R internalization; instead, it altered AT2R trafficking and significantly inhibited FRET between the two receptors (Fig. 4a,b), suggesting that the AT2R signaling cascade is also involved, albeit in part, in the conformational change of the receptor heterodimer. These data collectively indicate that signals from both receptors are indispensable for the conformational change in the receptor heterodimer; however, receptor dimerization itself can be induced by ligand binding to one receptor, as revealed by co-immunoprecipitation assay results (Fig. 2). Therefore, taken together with results shown in Fig. 1, the conformational change appears to correlate with the functional association and the co-internalization of the receptors.

### AT2R phosphorylation by PKC is required for the functional association between AT1R and AT2R

**Figure 5:**
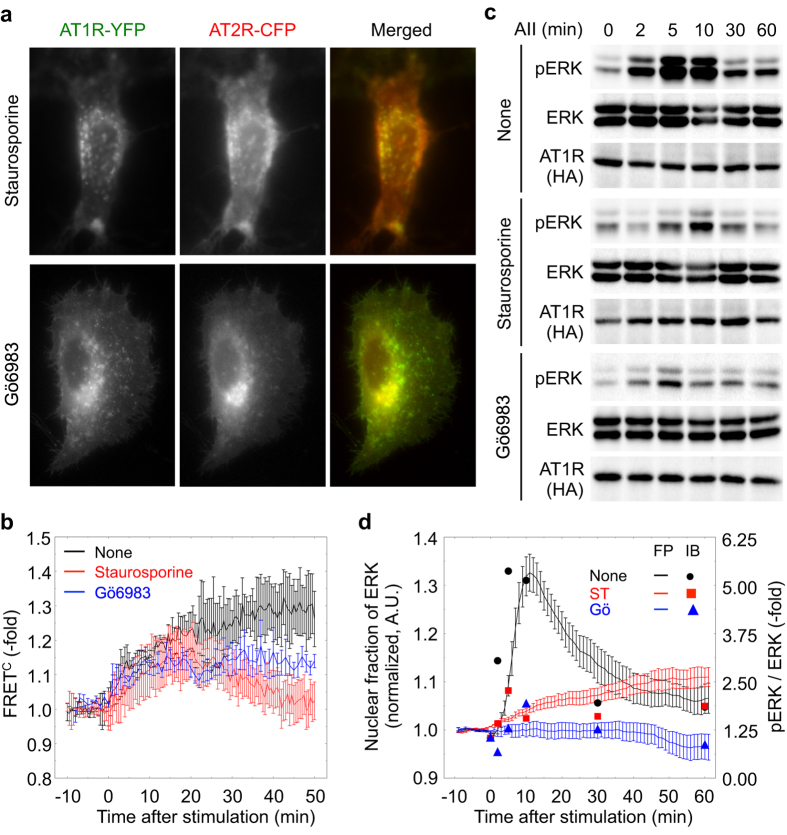
PKC activity is required for concomitant receptor internalization. (**a,b**) HeLa cells were transfected with AT1R-YFP and AT2R-CFP expression vectors. After 24 h, the cells were pretreated with a PKC inhibitor (either staurosporine or Gö6983) or left untreated for 30 min. The cells were then subjected to fluorescence microscopy and simulated by AII. Representative images are shown (**a**). The time courses of FRET^C^ in the presence or absence of staurosporine and Gö6983 are shown (**b**). Error bars indicate the s.e.m. (n = 3). (**c**) HeLa cells were transfected with AT1R expression vectors or control vectors. After 24 h, the cells were pretreated with either staurosporine or Gö6983 or were left untreated and then exposed to AII. The ERK phosphorylation status was evaluated by immunoblotting. (**d**) HeLa cells expressing AT1R and eqFP650-ERK were incubated in the presence or absence of the PKC inhibitors and were monitored by fluorescence microscopy, with exposure to AII at time 0. The nuclear ERK ratio (FP) and the intensity of the bands (in c; IB) were plotted over time. Error bars indicate the s.e.m. (n = 4).

Because PKC is a well-known downstream effector of AT1R^2^,^15^, we next investigated the effects of the PKC inhibitors staurosporine and Gö6983 on receptor dynamics. Whereas inhibitor treatment did not affect AT1R internalization, AT2R did not traffic with AT1R to the endosomes (Fig. 5a). In addition, whereas the PKC inhibitors displayed no effect on the FRET signal between the two receptors until 20 min after AII stimulation, it was significantly decreased from 20 min onward in the presence of the PKC inhibitors (Fig. 5b). These data indicate that PKC activation is indispensable for the functional association (conformational change in the receptor dimer) between AT1R and AT2R and the subsequent co-internalization of the dimer.

**Figure 6:**
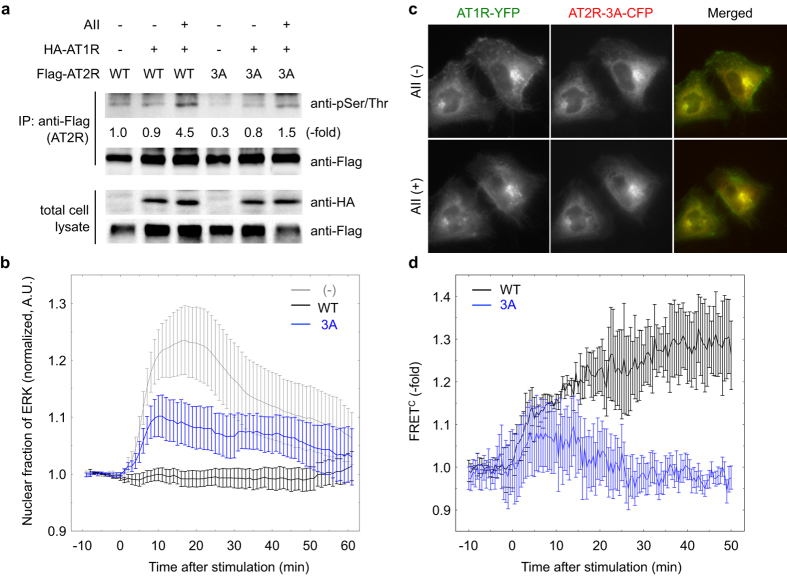
AT2R phosphorylation by PKC is required for the AT2R-mediated inhibition of ERK activation. (**a**) HeLa cells transfected with the expression vectors indicated at the top were serum starved for 4 h and stimulated by AII for 10 min. The cells were then lysed in lysis buffer and immunoprecipitated with an anti-FLAG antibody, followed by immunoblotting using an anti-phosphoserine/phosphothreonine (pSer/Thr) or anti-FLAG antibody. An aliquot of total cell lysate was also analyzed by immunoblotting. (**b**) HeLa cells were transfected with expression vectors for eqFP650-ERK, AT1R-YFP, and either AT2R-CFP or AT2R-3A-CFP. After 24 h, the cells were subjected to fluorescence microscopy and simulated by AII at time 0. The nuclear ERK ratio was plotted over time. Error bars indicate the s.e.m. (n = 3). (**c,d**) HeLa cells were transfected with AT1R-YFP and AT2R-3A-CFP expression vectors. After 24 h, the cells were subjected to fluorescence microscopy and simulated by AII. Representative images are shown (**c**). The time courses of FRET^C^ between AT1R and either AT2R or AT2R3A are shown (**d**). Error bars indicate the s.e.m. (n = 4).

Finally, we evaluated the effect of PKC on the AT2R-mediated inhibition of AT1R signaling. However, the utilization of PKC inhibitors might not be suitable because PKC is reported to mediate AT1R-dependent ERK activation^16^. Indeed, treatment with staurosporine or Gö6983 inhibited the phosphorylation and nuclear translocation of ERK caused by AII stimulation in cells expressing AT1R (Fig. 5c,d). Staurosporine treatment also inhibited ERK phosphorylation induced by AII-stimulated AT1R in 293T cells (Supple. Fig. S5a). Alternatively, we found three possible phosphorylation sites in the C-terminal region of AT2R through sequence analysis. Phosphorylation of AT2R was indeed triggered by AII-AT1R signaling, as revealed by immunoblot analysis of AT2R immunoprecipitates using anti-phosphoserine/phosphothreonine antibodies (Fig. 6a, lane 3). Given that this phosphorylation could be observed even in the presence of PD123319, AT2R phosphorylation might be totally dependent on AII binding on AT1R (Suppl. Fig. S5b). This result encouraged us to generate an AT2R mutant in which all three serine residues were substituted with alanine (AT2R-3A). AT1R signaling-induced phosphorylation of the AT2R mutant was inhibited compared with that of wild-type AT2R (Fig. 6a, lane 6), and the inhibitory effect on AT1R-mediated ERK activation was substantially diminished (Fig. 6b; Suppl. Fig. S5c). This inability of the AT2R mutant to suppress ERK activation was not affected by treatment with the AT2R inhibitor PD123319 (Suppl. Fig. S5d). Although the AT2R-3A mutant was not internalized with AT1R, it did colocalize with AT1R in subcellular compartments other than endosomes, including the plasma membrane and the perinuclear region (Fig. 6c). Furthermore, few FRET signals were observed between AT2R-3A and AT1R following exposure to AII (Fig. 6d). Taken together, these results indicate that the AII-dependent, AT1R-PKC-mediated phosphorylation of AT2R might be critical for hindering AT1R signaling by AT2R.

## Discussion

AT1R activates many downstream effectors, including ERK, which is reported to be a major regulator of AII-induced cardiovascular diseases^17^. ERK activation by AT1R is mediated by at least three different signaling pathways in a cell-context-specific manner: PKC-dependent activation, β-arrestin-dependent activation, and matrix metalloprotease and subsequent HB-EGF shedding-dependent activation^18^. Among these pathways, the first two have been extensively investigated in AT1R-expressing cells and shown to produce distinct outcomes in different subcellular compartments. For example, the PKC-dependent pathway mainly functions at the plasma membrane and modulates gene expression, whereas the β-arrestin-dependent cascade is involved in endocytosis and is dispensable for gene regulation^19^. Our findings suggest that AT2R might regulate these pathways differently because the conformational changes detected by the FRET signal occur mainly in the internalized receptors (Fig. 3c). As opposed to the receptor heterodimerization by itself, which could be induced by ligand binding to one of the receptors, the conformational change required for ligand binding to both receptors and PKC activity. These signal modulations might affect the AT1R signaling output, including AT1R-induced vasoconstrictive actions.

In general, it is widely accepted that AII counteracts AT1R-mediated vasoconstriction *in vivo*. Treatment with the AT2R inhibitor PD123319 enhances the AII responsiveness in the rat thoracic aorta after pressure overload^20^. In spontaneously hypertensive rats (SHRs), a lack of AT2R function results in enhanced coronary constriction mediated by AII^21^. Moreover, upon AT1R inhibition, AII evokes a hypotensive response, which is eliminated by AT2R antagonist treatment^22^. Genetic approaches also support these pharmacological characterizations of AT2R. Mice deficient in *At2r* (AT2R-KO), which develop normal blood pressure in the resting state, display enhanced vasoconstriction after AII injection^23^. Consistently, the AT1-mediated vasoconstrictive effect induced by the chronic infusion of AII is not observed in transgenic mice overexpressing AT2R^24^. However, AT2R may not counteract the function of AT1R in the heart. AII stimulates myocardial hypertrophy and fibrosis in AT2R-KO mice to levels comparable to those in wild-type mice^25^. Therefore, AT2R counteracts the function of AT1R in a cell-context-specific manner, although the detailed mechanisms underlying the difference have yet to be elucidated.

One possible mechanism for such cell type-specific AT1R signaling inhibition by AT2R might be ascribed to the relative expression of the receptors. In most adult tissues, AT2R levels, which are much lower than AT1R levels under physiological conditions, are increased upon exposure to environmental cues that activate AII-AT1R signaling^6^,^26^. Because the function of AII can be altered by the balance of receptor subtype abundances^6^,^27^, the extent of AT2R induction might be important for the modulation of AII signaling by AT2R. In particular, the amount of heterodimer, which was shown to be important for signaling modulation in this study, has been shown to be much smaller than the amount of AT1R and AT2R homodimers^28^. We can therefore postulate that AT2R induction at levels adequate to form an abundance of receptor heterodimers might be critical for the inhibition of AT1R signaling by AT2R.

As we clearly demonstrated, AT2R accumulated with AT1R in the endosomes after AII stimulation. In a previous report, the uptake of AT2R was shown to occur only in the presence of promyelocytic leukemia zinc finger protein (PLZF)^29^. Because the effects of AT2R are physiologically opposite from those of AT1R signaling and this synchronous association occurs immediately after AII stimulation, the functional interaction between AT1R and AT2R might be necessary for an acute response to extracellular environmental changes. However, it has also been proposed that AT2R can counteract AT1R signaling in a manner independent of receptor heterodimerization^28^. This raises the possibility that the mode of action of AT2R for AT1R inhibition is also cell-context specific. Moreover, AT2R was also shown to form a stable, functional heterodimer with bradykinin B2 receptor. In this case, AT2R contributes to the non-cell-autonomous inhibition of AT1R signaling through the enhanced production of nitric oxide^30^. Therefore, AII-AT1R signaling might be stringently regulated by the diverse molecular actions of AT2R.

**Figure 7:**
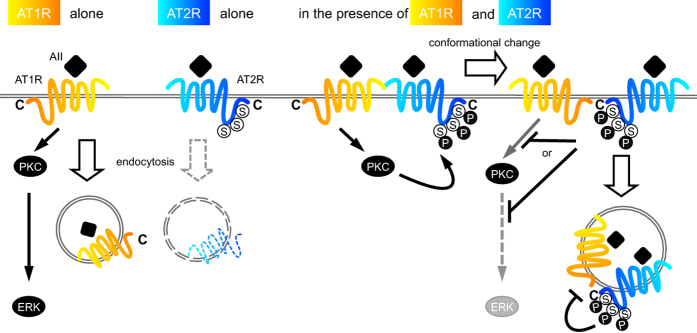
Schematic representation of the PKC-mediated inhibition of AT1R signaling by AT2R. AT1R stimulation by AII induces ERK phosphorylation via PKC and the internalization of AT1R *per se*, whereas AT2R in the absence of AT1R remains at the plasma membrane even after stimulation. In the presence of both receptors, AII stimulation induces the phosphorylation of AT2R at the C-terminus via PKC, the conformational change in receptor-receptor heterodimers, and the concomitant internalization of the receptors. This process is required for the AT2R-mediated inhibition of ERK phosphorylation by AT1R.

PKC is a crucial downstream effector of the AT1R signaling pathway. Paradoxically, PKC has been reported to be indispensable for AT1R endocytosis^31^. Our results show that AT2R internalization is dependent on AT1R and PKC, indicating that both receptors are internalized by distinct molecular mechanisms (Fig. 7). Given that the previous study demonstrated that Ser354 of AT2R is phosphorylated by PKC^32^, the phosphorylation of this amino acid residue by PKC might be necessary for AII-dependent AT2R internalization. Studies on the detailed molecular mechanism of PKC-dependent AT2R internalization are now in progress.

## Methods

### Expression plasmids

Human AT1R and AT2R cDNAs were obtained by conducting RT-PCR of the total RNA from human umbilical vein endothelial cells using the following primers: AT1R forward primer, 5′-CCGAATTCACCATGTACCCATACGATGTTCCAGATTACGCTATTCTCAACTCTTCTACTGAAG-3′; AT1R reverse primer, 5′-CCAGATCTCTACAGATCCTCTTCAGAGATGAGTTTCTGCTCGCGGCCGCACTCAACCTCAAAACATGGTG-3′; AT2R forward primer, 5′-CCGAATTCACCATGGACTACAAAGACGATGACGACAAGAAGGGCAACTCCACCCTTGC-3′; and AT2R reverse primer, 5′-CCAGATCTCTACAGATCCTCTTCAGAGATGAGTTTCTGCTCGCGGCCGCAAGACACAAAGGTCTCCATTTC-3′. These primers were designed for the insertion of HA- and FLAG-tags after the initial methionine, as previously described^33^. The resulting PCR products were cloned into the pCR-BluntII-TOPO vector (Invitrogen Corp., Carlsbad, CA, USA). To obtain the HA-AT1R-YFP and FLAG-AT2R-CFP plasmids, HA-AT1R and FLAG-AT2R were subcloned into the *Eco*RI and *Not*I sites of the pCXN2-YFPC and pCXN2-CFPC vectors, respectively^34^. To generate the phosphorylation-deficient AT2R mutant in which the three serine residues at 352–354 a.a. were changed to alanine, its coding sequence was amplified by PCR using the AT2R forward primer, shown above, and the AT2R-AAA reverse primer, 5′- CCGCGGCCGCAAGACACAAAGGTCTCCATTTCTCTAAGGGCGGCGGCTTTCCGGCAAGACATACTCTC-3′. The resulting PCR product was subcloned into the *Eco*RI and *Not*I sites of pCXN2-CFPC vectors. cDNA for *Entacmaea quadricolor* eqFP650 (TurboFP650)^10^ was purchased from Evrogen (Moscow, Russia) and amplified by PCR using the following primers: eqFP650 forward primer, 5′-AAGAATTCATGGGAGAGGATAGCGAG-3′; eqFP650 reverse primer, 5′-TTAGATCTGCTGTGCCCCAGTTTG-3′. The resulting PCR product was subcloned into a pCMV-derived vector to obtain pFX-eqFP650. *Rattus* ERK2 cDNA was amplified by PCR using the following primers: ERK2 forward primer, 5′-GGCTCGAGATGGCGGCGGCGGCGGCGGCG-3′; ERK2 reverse primer, 5′-CCGCGGCCGCTTAAGATCTGTATCCTGGC-3′. The resulting PCR product was subcloned into the *Xho*I and *Not*I sites of the pFX-eqFP650 vector to obtain pFX-eqFP650-ERK2. All constructs were confirmed by sequencing analysis.

### Reagents and antibodies

AII, LPA, and PD123319 were obtained from Sigma (St. Louis, MO, USA). EGF and losartan were purchased from PeproTech (Rocky Hill, NJ, USA) and LKT Laboratories (St. Paul, MN, USA), respectively, and staurosporine and AG1478 were purchased from Calbiochem (Darmstadt, Germany). Hoechst 33342 and Gö6983 were obtained from Invitrogen (Carlsbad, CA, USA) and Cayman Chemical (Ann Arbor, MI, USA), respectively. Antibodies against FLAG and HA were purchased from Stratagene (La Jolla, CA, USA) and Roche (Indianapolis, IN, USA), respectively, and antibodies against ERK1/2 and phospho-ERK1/2 were purchased from Cell Signaling Technology (Beverly, MA, USA). A series of antibodies against phosphorylated serine and threonine (Phosphoserine detection set and Phosphothreonine detection set) were obtained from Enzo Life Sciences (Farmingdale, NY, USA).

### Cell culture and transfection

HeLa cells were cultured in Dulbecco’s modified Eagle’s medium (DMEM, Sigma) supplemented with 10% FBS. Expression plasmids were introduced into cells with FuGENE HD (Roche), according to the manufacturer’s protocol.

### Immunoblotting and immunoprecipitation assay

Immunoblotting was performed as previously described, with some modifications^33^. Briefly, cells were lysed in lysis buffer [20 mM HEPES, pH 7.5, 50 mM NaCl, 0.3% digitonin, 10% glycerol, 5 mM MgCl_2_, 3 mM EGTA, 0.1 mM Na_3_VO_4_, 20 mM NaF and complete EDTA-free protease inhibitor (Roche, Indianapolis, IN, USA)] for 20 min on ice and clarified by microcentrifugation. Supernatants were subjected to SDS-PAGE, and the separated proteins were transferred to polyvinylidene difluoride membranes (Bio-Rad, Hercules, CA, USA). The membrane was incubated with a primary antibody followed by a peroxidase-labeled secondary antibody. Signals were developed by ECL Western Blotting Detection Reagent (GE Healthcare, Little Chalfont, UK) and detected using an LAS-1000 UV mini image analyzer (FUJIFILM, Tokyo, Japan). The intensities of the bands were quantitated using the associated software. Note that samples were incubated with SDS sample buffer at 50 °C but were not boiled to detect AT1R and AT2R while preventing their aggregation.

HeLa cells expressing HA-AT1R-YFP and Flag-AT2R-CFP were lysed in NP-40 lysis buffer [10 mM Tris-HCl, pH 7.4, 150 mM NaCl, 5 mM EDTA, 0.5% NP-40, 10% glycerol, 1 mM Na_3_VO_4_, and complete EDTA-free protease inhibitor (Roche)] and precipitated with an anti-FLAG antibody with protein A-Sepharose beads. Proteins bound to the beads were separated by SDS-PAGE and detected by immunoblotting.

### Fluorescence microscopy

Intermolecular FRET analysis was performed as previously described^34^,^35^. Briefly, HeLa cells were cultured on glass-bottom, 35-mm tissue culture dishes (Asahi Techno Glass Co., Tokyo, Japan) and transfected with expression vectors for fluorescent protein-tagged fusion proteins. The fluorescence imaging workstation for the multicolor time-lapse imaging consisted of an Olympus IX71 inverted microscope equipped with a cooled charge-coupled device (Cool-SNAPHQ, Roper Scientific, Trenton, NJ), excitation and emission filter wheels (MAC 5000, Ludl Electronic Products, Hawthorne, NY), and a Xenon 75-W light source, all controlled by MetaMorph software (Universal Imaging, Downingtown, PA). Images were recorded every 30 sec for up to 1 h. Starting after 10 min, the cells were stimulated with AII. At each time point, fluorescence images were sequentially acquired through the YFP, CFP, and FRET filter channels. The filter sets used were YFP (excitation, 500-25 nm; emission, 535-26 nm), CFP (excitation, 440-21 nm; emission, 480-30), and FRET (excitation, 440-21 nm; emission, 535-26 nm). An XF2034 (455 DRLP) dichroic mirror (Omega Optical, Brattleboro, VT) was used. Images were acquired using the 4 × 4 binning mode and 100 to 200 ms integration times. The background was subtracted from the raw images before FRET calculations. Corrected FRET (FRET^C^) was calculated on a pixel-by-pixel basis for the entire image by using the following equation: FRET^C^ = FRET-(0.5 × CFP)-(0.02 × YFP), where FRET, YFP, and CFP correspond to the background-subtracted images of cells co-expressing CFP and YFP acquired through the FRET, YFP, and CFP channels, respectively. The bleed-through fractions of CFP and YFP fluorescence were 0.5 and 0.02, respectively, through the FRET channel. FRET^C^ images are presented in pseudocolor mode. A mask image for the entire cell was created based on the YFP fluorescence intensity, and FRET^C^ values in the region were quantitated using MetaMorph software.

Fluorescence images of eqFP650-ERK and Hoechst-stained nuclei were acquired as described above, except different filter sets were used (for Hoechst, excitation: 400-15 nm, emission: 480-30 nm; for eqFP650, excitation: 535-30 nm, emission: 692-40 nm). A mask image for the nuclear region was created based on the Hoechst fluorescence intensity, and the fluorescence intensities of eqFP650-ERK in the nucleus and the entire cell were quantitated to measure ERK activity.

## Additional Information

**How to cite this article**: Inuzuka, T. *et al*. Attenuation of ligand-induced activation of angiotensin II type 1 receptor signaling by the type 2 receptor via protein kinase C. *Sci. Rep.* **6**, 21613; doi: 10.1038/srep21613 (2016).

## Supplementary Material

Supplementary Information

Supplementary Video S1

Supplementary Video S2

Supplementary Video S3

Supplementary Video S4

Supplementary Video S5

Supplementary Video S6
