# Supplementary material for: Attenuation of ligand-induced activation of angiotensin II type 1 receptor signaling by the type 2 receptor via protein kinase C
Source: Sci Rep. 2016 Feb 9;6:21613. doi: 10.1038/srep21613 (PMC4746669; doi:10.1038/srep21613)
Supplement: Supplementary Information [file srep21613-s1.pdf]

Attenuation of ligand-induced angiotensin II type 1 receptor signaling by the type 2 receptor via protein kinase C

Takayuki Inuzuka[1], Yoichiro Fujioka[1], Masumi Tsuda[2], Mari Fujioka[1], Aya O. Satoh[1], Kosui Horiuchi[1], Shinya Nishide[1], Asuka Nanbo[1], Shinya Tanaka[2] & Yusuke Ohba[1]

[1]Department of Cell Physiology, Hokkaido University Graduate School of Medicine, Sapporo 060-8638, Japan
[2]Department of Cancer Pathology, Hokkaido University Graduate School of Medicine, Sapporo 060-8638, Japan

Supplementary Figures S1–S5

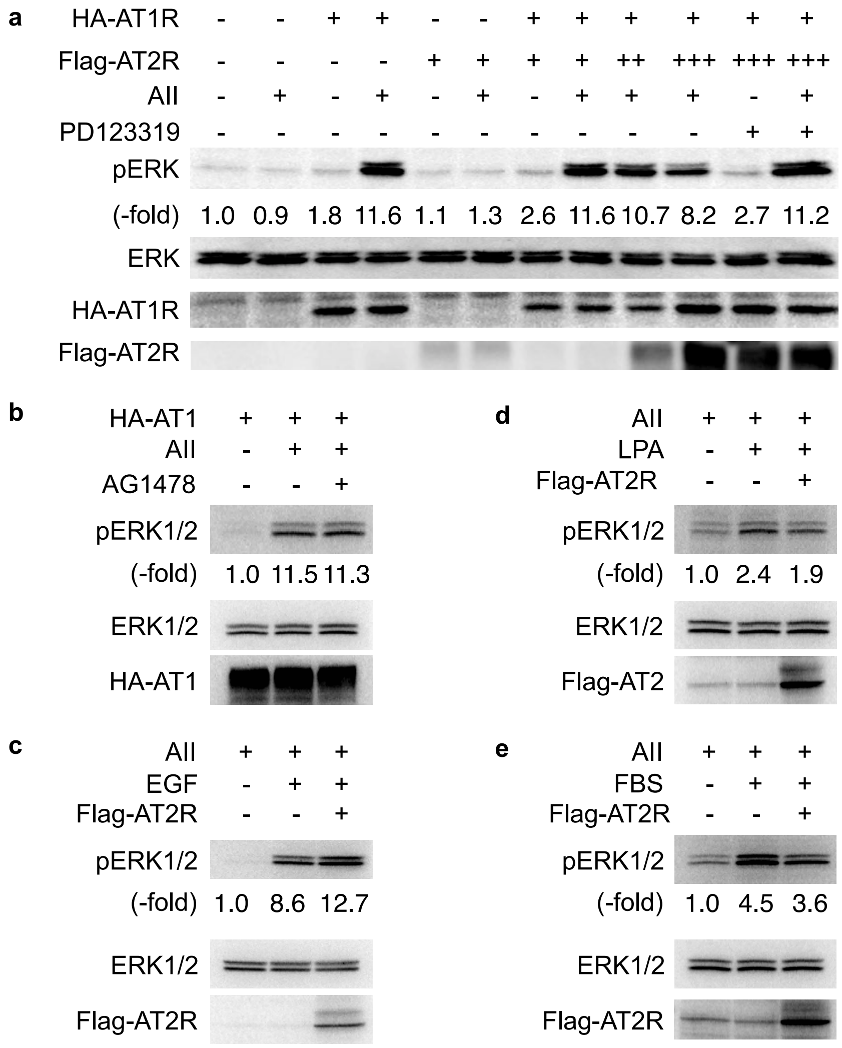

Supplementary Figure S1. AT2R disturbs AT1R signaling (pertaining to Fig. 1). (a) HEK293T cells were transfected with expression vectors for the proteins indicated at the top. After 24 h, the cells were serum-starved for 4 h, stimulated by AII or left untreated, and then subjected to SDS-PAGE. The phosphorylation status of ERK was determined by immunoblotting using a phospho-specific antibody against ERK. Expression levels of ERK, AT1R and AT2R were also evaluated using antibodies to ERK, HA, and FLAG. (b) HEK293T cells expressing AT1R were treated as indicated on the top and subjected to immunoblotting. (c–e) HEK293T cells transfected with the AT2R expression vector (the right lanes) or the control vector (left two lanes) were pretreated with AII for 4 h and stimulated by EGF (c), LPA (d), and fetal bovine serum (FBS, e) as indicated.

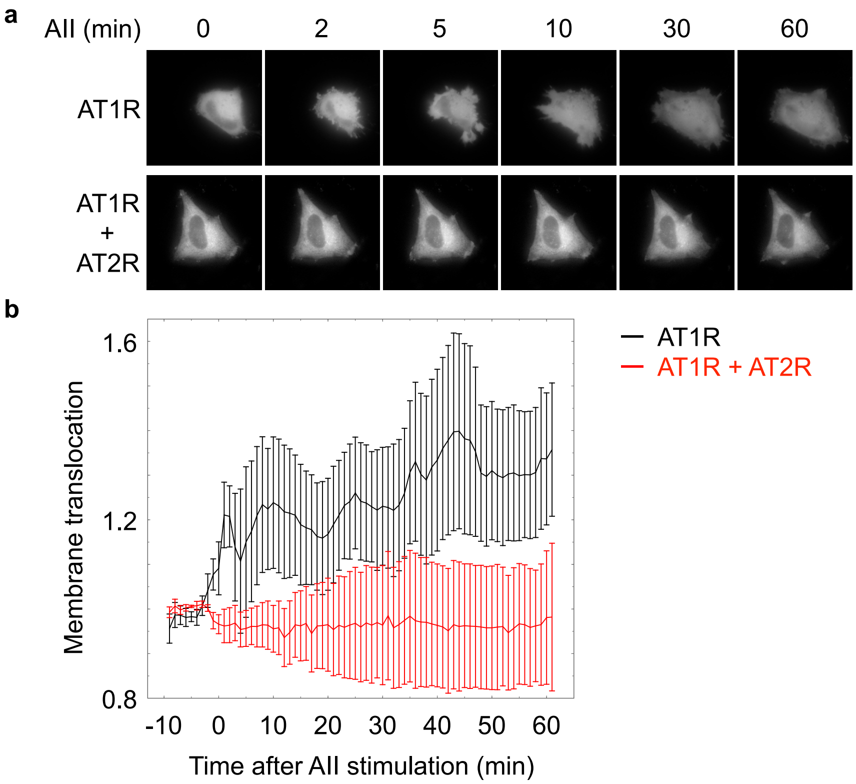

Supplementary Figure S2. AT2R inhibits the AT1R-dependent membrane translocation of c-Raf1. HeLa cells transfected with an expression vector for eqFP650-c-Raf1, along with expression vectors for AT1R alone or for both AT1R and AT2R, were observed by fluorescence time-lapse microscopy. Starting at 10 min, the cells were stimulated by AII. Representative images at indicated time points are shown (a). The extent of c-Raf1 membrane localization was assumed as the ratio of fluorescence intensities in the nuclear and cytoplasmic regions and was plotted over time (b). Error bars indicate the s.e.m. ($n = 4$).

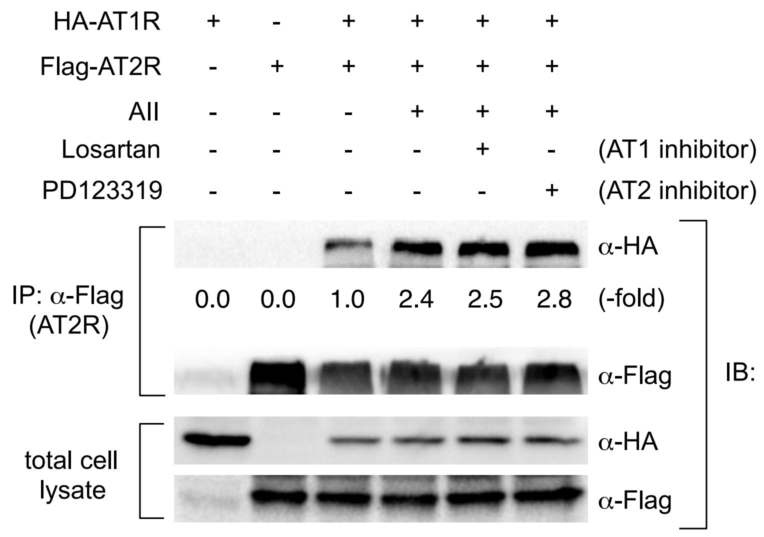

Supplementary Figure S3. AT2R interacts with AT1R (pertaining to Fig. 2). 293T cells transfected with the expression vectors indicated at the top were serum-starved, pre-treated with the AT1R specific inhibitor losartan or the AT2R specific inhibitor PD123319, and stimulated by AII. The cells were lysed in lysis buffer and immunoprecipitated with an anti-FLAG antibody followed by immunoblotting using an anti-HA or anti-FLAG antibody. An aliquot of total cell lysate was also analyzed by immunoblotting.

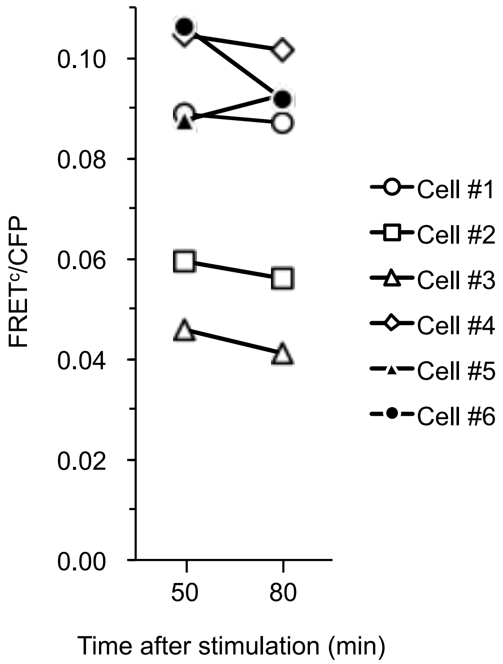

Supplementary Figure S4. FRET between AT1R-YFP and AT2R-CFP (pertaining to Fig. 3d). HeLa cells transfected with expression vectors for AT1R-YFP or AT2R-CFP were observed with fluorescence microscopy. The corrected FRET (FRETc) per CFP ratio at 50 and 80 min after AII stimulation in each cell were plotted.

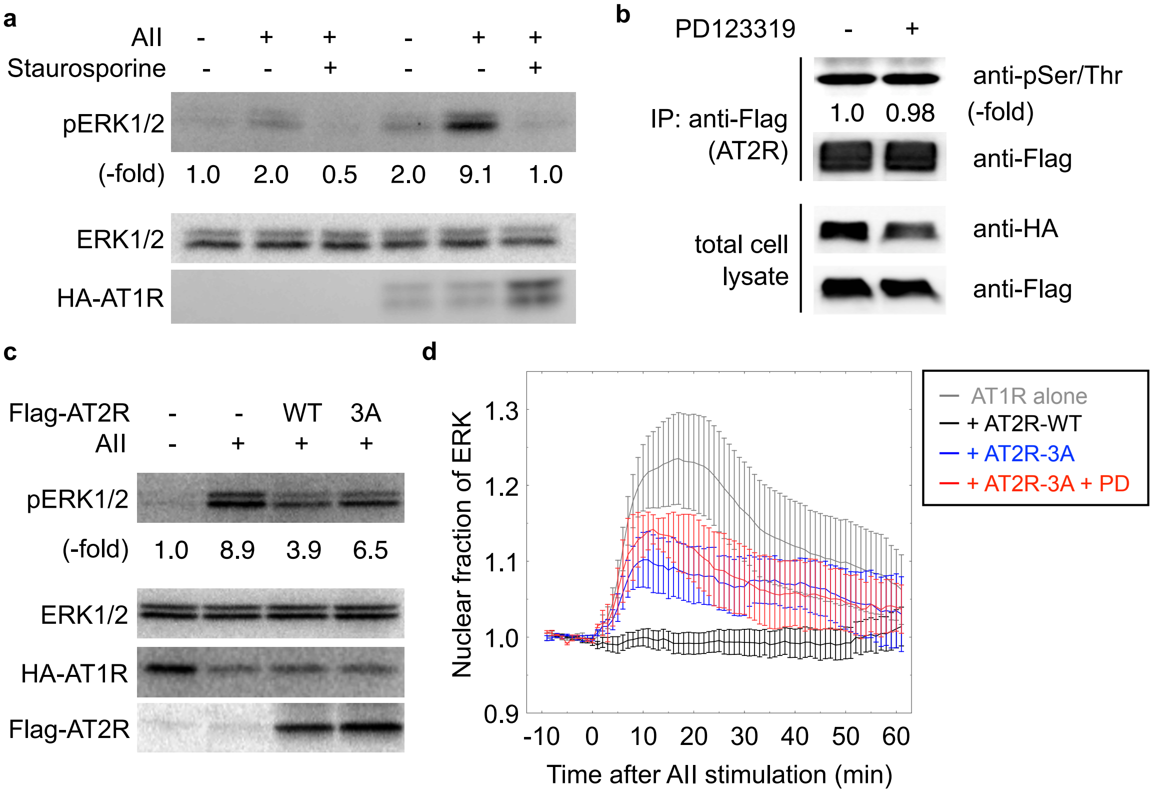

Supplementary Figure S5. PKC activity is required for the concomitant internalization of receptors and AT2R-mediated inhibition of ERK activation (pertaining to Figs. 5 and 6). (a) HEK293T cells were transfected with expression vectors for AT1R or control vectors. After 24 h, the cells were pretreated with staurosporine or left untreated and then exposed to AII. The phosphorylation status of ERK was evaluated by immunoblotting. (b) 293T cells transfected with the expression vectors indicated at the top were incubated in the presence or absence of the AT2R inhibitor PD123319, serum starved for 4 h, and stimulated by AII for 10 min. The cells were then analyzed as in Fig. 6a. (c) HEK293T cells were transfected with expression vectors for AT1R alone or with those for AT2R or AT2R-3A as indicated at the top. The cells were exposed to AII and their ERK phosphorylation status was evaluated. (d) HeLa cells were transfected with expression vectors for eqFP650-ERK, AT1R-YFP, and AT2R-3A-CFP. After 24 h, the cells were treated with the AT2R inhibitor PD123319 for 4 h and were then subjected to fluorescence microscopy, with exposure to AII at time 0. The nuclear ERK ratio was plotted over time. Error bars indicate the s.e.m. (n = 3). The data from Fig. 6c are also shown here for comparison.

Supplementary Movies S1–S6

Supplementary Movie S1. Time-lapse imaging of HeLa cells expressing AT1R. Cells transfected with an AT1R-YFP expression vector were observed by fluorescence time-lapse microscopy. Starting at 10 min, the cells were stimulated by AII (pertaining to Fig. 3a).

Supplementary Movie S2. Time-lapse imaging of HeLa cells expressing AT2R. Cells transfected with an AT2R-CFP expression vector were observed with fluorescence time-lapse microscopy. Starting at 10 min, the cells were stimulated by AII (pertaining to Fig. 3a).

Supplementary Movie S3. Time-lapse imaging of HeLa cells expressing AT1R and AT2R. Cells transfected with AT1R-YFP and AT2R-CFP expression vectors were observed with fluorescence time-lapse microscopy. Starting at 10 min, the cells were stimulated by AII. YFP images are shown (pertaining to Fig. 3b).

Supplementary Movie S4. Time-lapse imaging of HeLa cells expressing AT1R and AT2R. Cells transfected with AT1R-YFP and AT2R-CFP expression vectors were observed with fluorescence time-lapse microscopy. Starting at 10 min, the cells were stimulated by AII. CFP images are shown (pertaining to Fig. 3b).

Supplementary Movie S5. Time-lapse imaging of HeLa cells expressing AT1R and AT2R. Cells transfected with AT1R-YFP and AT2R-CFP expression vectors were observed with fluorescence time-lapse microscopy. Starting at 10 min, the cells were stimulated by AII. Merged images are shown (pertaining to Fig. 3b).

Supplementary Movie S6. Time-lapse imaging of HeLa cells expressing AT1R and AT2R. Cells transfected with AT1R-YFP and AT2R-CFP expression vectors were observed with fluorescence time-lapse microscopy. Starting at 10 min, the cells were stimulated by AII. Reconstructed $FRET^C$ images are shown in pseudocolor mode (pertaining to Fig. 3c).
